# Application of Association Mapping to Understanding the Genetic Diversity of Plant Germplasm Resources

**DOI:** 10.1155/2008/574927

**Published:** 2008-06-08

**Authors:** Ibrokhim Y. Abdurakhmonov, Abdusattor Abdukarimov

**Affiliations:** Center of Genomic Technologies, Institute of Genetics and Plant Experimental Biology, Academy of Sciences of Uzbekistan, Yuqori Yuz, Qibray region, Tashkent district 702151, Uzbekistan

## Abstract

Compared to the conventional linkage mapping, linkage disequilibrium (LD)-mapping, using the nonrandom associations of loci in haplotypes, is a powerful high-resolution mapping tool for complex quantitative traits. The recent advances in the development of unbiased association mapping approaches for plant population with their successful applications in dissecting a number of simple to complex traits in many crop species demonstrate a flourish of the approach as a “powerful gene tagging” tool for crops in the plant genomics era of 21st century. The goal of this review is to provide nonexpert readers of crop breeding community with (1) the basic concept, merits, and simple description of existing methodologies for an association mapping with the recent improvements for plant populations, and (2) the details of some of pioneer and recent studies on association mapping in various crop species to demonstrate the feasibility, success, problems, and future perspectives of the efforts in plants. This should be helpful for interested readers of international plant research community as a guideline for the basic understanding, choosing the appropriate methods, and its application.

## 1. INTRODUCTION

The level of the genetic diversity is pivotal for world food security and survival of human civilization on earth. Historically, humans exploited plant species for their livelihoods that resulted in domestication of many of them as improved cultivars to produce food for the better supply of the human diet [1]. Presently, out of 150 plant species cultivated in agriculture, twelve provide about 75% of human food and four produce 50% of human diet [2]. According to Food and Health Organization report, ~800 million people in the developing countries are suffering from food deficiency [3] that underlies an attention to improve agricultural production to eliminate or, at least, reduce the feeding problems.

The narrow genetic base of modern crop cultivars is the serious obstacle to sustain and improve crop productivity due to rapid vulnerability of genetically uniform cultivars by potentially new biotic and abiotic stresses [4]. However, plant germplasm resources worldwide, comprising of wild plant species, modern cultivars, and their crop wild relatives, are the important reservoirs of natural genetic variations, originated from a number of historical genetic events as a respond to environmental stresses and selection through crop domestication [1, 5]. The efficient exploiting these ex situ conserved genetic diversities is vital to overcome future problems associated with narrowness of genetic base of modern cultivars. However, many agriculturally important variations such as productivity and quality, tolerance to environmental stresses, and some of forms of disease resistance are controlled by polygenes and “multifactorial” that greatly depends on *genetic* × *environmental* (G × E) interactions [1, 6]. These complex traits are referred to as quantitative trait loci (QTLs), and it is challenging to identify QTLs based on only traditional phenotypic evaluation. Identification of QTLs of agronomic importance and its utilization in a crop improvement further requires mapping of these QTLs in a genome of crop species using molecular markers [1, 6]. This was the major breakthrough and accomplishment in many crops in “genomics era” since the end of the 20th century, and now extended to flourish in the 21st century.

In this review, we provide a brief description for the concept of genetic mapping; then, as a flourish of the crop genomics era, we thoroughly review one of the powerful genetic mapping tools for crops, linkage disequilibrium (LD)-based association study, as a high-resolution, broader allele coverage, and cost effective gene tagging approach in plant germplasm resources. This provides an opportunity to widely dissect and exploit existing natural variations for crop improvement.

## 2. GENETIC MAPPING OF CAUSATIVE VARIANTS

The main goal of genetic mapping is to detect neutrally inherited markers in close proximity to the genetic causatives or genes controlling the complex quantitative traits. Genetic mapping can be done mostly in two ways [1]: (1) using the experimental populations (also referred to as “biparental” mapping populations) that is called QTL-mapping as well as “genetic mapping” or “gene tagging,” and (2) using the diverse lines from the natural populations or germplasm collections that is called LD-mapping or “association mapping.” The details of the traditional QTL-mapping approach has recently been reviewed by Collard et al. [6], and further basic description of the approach here would be a redundant. For detailed concept, models and methodologies, problems, and perspectives of linkage analysis, readers are suggested refer to Liu [7] and Wu et al. [8]. Here, we briefly outline linkage mapping procedure for the sake of highlighting the merits of the alternative approach-association mapping.

So that such a linkage analysis can be done [6–8], firstly, the experimental populations such as *F*_2_, back cross (BC), double haploid (DH), recombinant inbred line (RIL), and near isogenic line (NIL) populations, derived from the genetic hybridization of two parental genotypes with an alternative trait of interest, need to be developed. Secondly, these experimental populations including a large number of progenies or lines are measured for the segregation of a trait of interest in the different environmental conditions. Thirdly, a set of polymorphic DNA markers, differentiating the parental genotypes and segregating in a mapping population, need to be identified and genotyped. For that, usual practice is that, first, the parental genotypes are screened, and if markers are polymorphic over the parents, then, all individuals of a mapping population are genotyped with these polymorphic molecular markers. Once genotypic data of a mapping population is ready, marker data is used to construct the framework linkage maps, representing the order (position) and linkage (a relative genetic distance in cM) of used molecular markers along the linkage groups or segments of particular chromosomes. This is accomplished through assessing of recombination rates between the marker loci. Consequently, these markers ordered along the linkage map are statistically correlated with phenotypic characteristics of individuals of a mapping population, and QTL regions affecting a trait of interest, along with closely positioned marker tags to that QTL, are identified.

One can imagine these linkage marker maps as a “road map,” marker tags as the labels directing to specific places, and QTLs to a community/neighborhood (with specific function) on the map [6]. The precision of QTL-mapping largely depends on the genetic variation (or genetic background) covered by a mapping population, the size of a mapping population, and a number of marker loci used. Once QTLs affecting a trait of interest accurately tagged using above-outlined approach, marker tags are the most effective tools in a crop improvement that allows the mobilization of the genes of interest from donor lines to the breeding material through marker-assisted selection (MAS). Although traditional QTL-mapping will continue being an important tool in gene tagging of crops, it is a “now classical approach” and overall is very costly [1, 9], and has low resolution with simultaneous evaluation of only a few alleles [10] in a longer research time scale. In linkage mapping, the major limitation, hampering the fine mapping, is associated with the availability of only a few meiotic events to be used that occurred since experimental hybridization in a recent past [11].

## 3. ASSOCIATION MAPPING AS AN ALTERNATIVE APPROACH

These limitations, however, can be reduced with the use of “association mapping” [1]. Turning the gene-tagging efforts from biparental crosses to natural population of lines (or germplasm collections), and from traditional QTL-mapping to linkage disequilibrium (LD)-based association study became a powerful tool in mapping of the genes of interest [12]. This leads to the most effective utilization of ex situ conserved natural genetic diversity of worldwide crop germplasm resources. LD refers to a historically reduced (nonequilibrium) level of the recombination of specific alleles at different loci controlling particular genetic variations in a population. This LD can be detected statistically, and has been widely applied to map and eventually clone a number of genes underlying the complex genetic traits in humans [13–16].

The advantages of population-based association study, utilizing a sample of individuals from the germplasm collections or a natural population, over traditional QTL-mapping in biparental crosses primarily are due to (1) availability of broader genetic variations with wider background for marker-trait correlations (i.e., many alleles evaluated simultaneously), (2) likelihood for a higher resolution mapping because of the utilization of majority recombination events from a large number of meiosis throughout the germplasm development history, (3) possibility of exploiting historically measured trait data for association, and (4) no need for the development of expensive and tedious biparental populations that makes approach timesaving and cost-effective [17–19].

**Figure 1:**
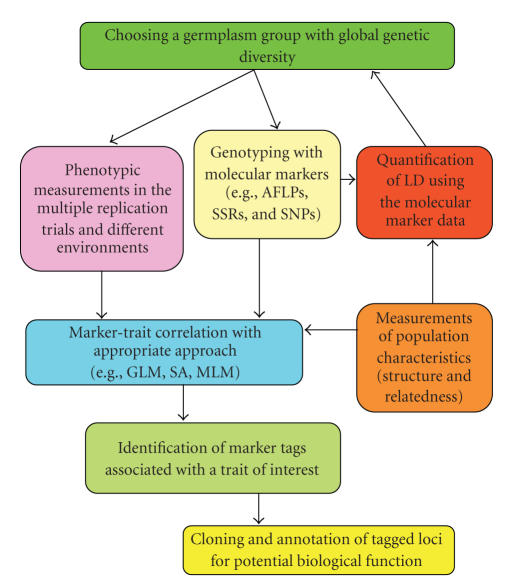
The scheme of association mapping for tagging a gene of interest using germplasm accessions. Note that the outlined scheme may vary based on population characteristics and methodology chosen for association study.

Although the overall approach of population-based association mapping in plants varies based on the methodology chosen (see below sections), assuming structured population samples, the performance of association mapping includes the following steps (see Figure 1): (1) selection of a group of individuals from a natural population or germplasm collection with wide coverage of genetic diversity; (2) recording or measuring the phenotypic characteristics (yield, quality, tolerance, or resistance) of selected population groups, preferably, in different environments and multiple replication/trial design; (3) genotyping a mapping population individuals with available molecular markers; (4) quantification of the extent of LD of a chosen population genome using a molecular marker data; (5) assessment of the population structure (the level of genetic differentiation among groups within a sampled population individuals) and kinship (coefficient of relatedness between pairs of each individuals within a sample); and (6) based on information gained through quantification of LD and population structure, correlation of phenotypic and genotypic/haplotypic data with the application of an appropriate statistical approach that reveals “marker tags” positioned within close proximity of targeted trait of interest. Consequently, a specific gene(s) controlling a QTL of interest can be cloned using the marker tags and annotated for an exact biological function (Figure 1). As a starting point for association mapping, it is important to gain knowledge of the patterns of LD for genomic regions of the “target” organisms and the specificity of the extent of LD among different populations or groups to design and conduct unbiased association mapping [20, 21]

## 4. LINKAGE DISEQUILIBRIUM (LD)

### 4.1. Concept of LD

Genetic linkage generally refers to coinheritance of different loci within a genetic distance on the chromosome. There are two terms used in population genetics, linkage equilibrium (LE), and linkage disequilibrium (LD) to describe linkage relationships (co-occurrence) of alleles at different loci in a population. LE is a random association of alleles at different loci and equals the product of allele frequencies within haplotypes, meaning that at random combination of alleles at each locus its haplotypes (combination of alleles) frequency has equal value in a population. In contrast, LD is a nonrandom association of alleles at different loci, describing the condition with nonequal (increased or reduced) frequency of the haplotypes in a population at random combination of alleles at different loci. LD is not the same as linkage, although tight linkage may generate high levels of LD between alleles. Usually, there is significant LD between more distant sites or sites located in different chromosomes, caused by some specific genetic factors [9, 22–24] that will be discussed in below sections. Linkage disequilibrium also referred as “gametic phase disequilibrium” (GPD) or “gametic disequilibrium” (GLD) [11, 25] in the literature that describes the same nonrandom association of haplotypes within unrelated populations with a distantly shared ancestry, assuming Hardy-Weinberg equilibrium (HWE).

The concept of LD was first described by Jennings in 1917, and its quantification (*D*) was developed by Lewtonin in 1964. The simplified explanation of the commonly used LD measure, *D* or *D*′ (standardized version of *D*), is the difference between the observed gametic frequencies of haplotypes and the expected gametic haplotype frequencies under linkage equilibrium (*D* = P_AB_ − P_A_P_B_ = P_AB_P_ab_ − P_Ab_P_aB_) [26]. Besides *D*, a various different measures of LD (*D*′, *r* ^2^, *D* ^2^, D*, *F*, *X* (2), and *δ*) have been developed to quantify LD [25, 27–29]. The detail formulae and description of LD quantification was well explained by a number of review papers [10, 25, 26] with a number of hypothetical scenarios for LD and LE. The merits, sensitivity, comparison, appropriate statistical tests, and calculation methodology for these LD measures with the utilization of biallelic or multiallelic loci have been extensively described in the literature in detail [10, 26, 30, 31], and have recently been reviewed by Gupta et al. [25]. Hence here we highlight only some of key utility properties of LD measures to provide a brief understanding the merits of LD in association mapping.

Choosing the appropriate LD measures really depends on the objective of the study, and one performs better than other in particular situations and cases; however, *D*′ and *r* ^2^ is the most commonly used measures of LD [25, 26]. *D*′ is informative for the comparisons of different allele frequencies across loci and strongly inflated in a small sample size and low-allele frequencies; therefore, intermediate values of *D*′ is dangerous for comparative analyses of different LD studies and should be verified with the *r* ^2^ before using for quantification of the extent of LD [26]. The *r* ^2^, the square of the correlation coefficient between the two loci have more reliable sampling properties than *D*′ with the cases of low allele frequencies [26]. The *r* ^2^ is affected by both mutation and recombination while *D*′ is affected by more mutational histories (it might indicate minimal historic recombination when high *D*′ values used) [10, 25, 26, 31]. Considering the objective, the most appropriate LD quantification measure needed for association mapping is *r* ^2^ that is also an indicative of marker-trait correlations [25, 26, 32]. The *r* ^2^ value varies from 0 to 1, and it will be equal to 1 when only two haplotypes are present. The *r* ^2^ value of equal to 0.1 (10%) or above considered the significant threshold for the rough estimates of LD to reveal association between pairs of loci [33].

It is noteworthy to briefly mention here that the estimation of above described GLD (commonly used in association mapping) between different loci ordered within gametes assumes that a targeted population or sampled germplasm is randomly mating and under HWE. Nevertheless, many natural populations violate HWE due to different genetic events (bottleneck, mutation, admixture, artificial selection, population structure, etc.) occurred in history of a population, and are under Hardy-Weinberg disequilibrium (HWD). A concept of “zygotic disequilibrium (ZLD)” was introduced for such a nonequilibrium population [34] that measures LD between different loci of gametes. ZLD, being defined as a deviation of joint zygotic frequencies from the expected values of zero zygotic associations [35, 36], has a power to measure nonrandom associations at both gametic and zygotic level [34, 37]. It shares the most of statistical properties of GLD [36], and the results of GLD and ZLD are mostly in agreement, yet ZLD detects more extensive LD than determined by GLD [37]. The statistical models of ZLD measures for biallelic and multilocus data, its application for natural populations, and inference the genetic and demographic events from the comparisons of GLD and ZLD results as well as implication for whole genome association studies (WGAs) were excellently addressed and described by a number of studies [35–37].

### 4.2. Calculation and visualization of LD: LD triangle and decay plots

LD can be calculated using available haplotyping algorithms [26]. One of such efficient methodology is the maximum likelihood estimate (MLE) using an expectation maximization algorithm [38]. Several computer software packages are available and can be utilized for calculation of LD using variety type of molecular markers. These software packages were extensively listed and described in the review by Gupta et al. [25].

**Figure 2:**
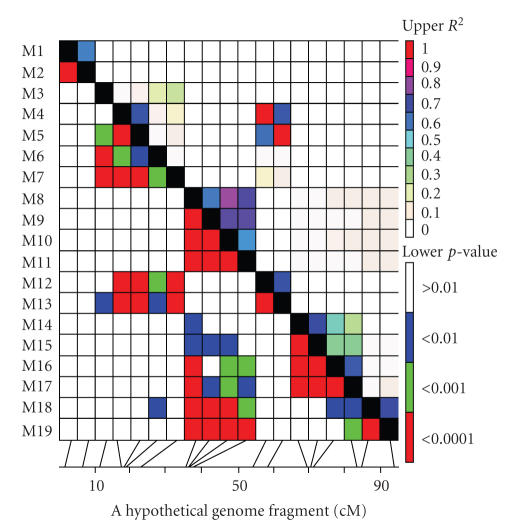
The TASSEL generated triangle plot for pairwise LD between marker sites in a hypothetical genome fragment, where pairwise LD values of polymorphic sites are plotted on both the *X*- and *Y*-axis; above the diagonal displays *r* ^2^ values and below the diagonal displays the corresponding *p*-values from rapid 1000 shuffle permutation test. Each cell represents the comparison of two pairs of marker sites with the color codes for the presence of significant LD. Colored bar code for the significance threshold levels in both diagonals is shown. The genetic distance scale for a hypothetical genome fragment was manually drawn. Note: this is for demonstration purposes only and does not have any real impact or correspond to any genomic fragment of an organism.

Graphical display of pairwise LD between two loci is very useful to estimate the LD patterns measured using a large number of molecular markers. Pairwise LD can be depicted as a color-code triangle plot (Figure 2) based on significant pairwise LD level (*r* ^2^, and *p*-value as well as *D*′) that helps to visualize the block of loci (red blocks) in significant LD. The large red blocks of haplotypes along the diagonal of the triangle plot indicate the high level of LD between the loci in the blocks, meaning that there has been a limited or no recombination since LD block formations. There is freely available specific computer software, “graphical overview of linkage disequilibrium” (GOLD) [39], to depict the structure and pattern of LD. Some other software packages measuring LD such as “Trait Analysis by aSSociation, Evolution and Linkage” (TASSEL) [33, 40] and PowerMarker [41] have also similar graphical display features. The strong block-like LD structures are of a great interest in association mapping which simplifies LD mapping efforts of complex traits [42]. LD blocks are very useful in association mapping when sizes are calculated, which suggest the needs for the minimum number of markers to efficiently cover the genome-wide haplotype blocks in association mapping.

**Figure 3:**
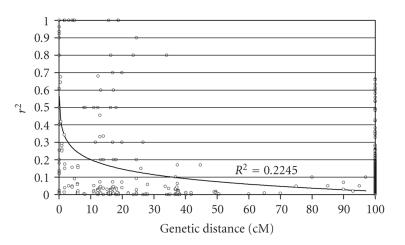
Linkage disequilibrium (LD) decay plot depicted from the LD values of a hypothetical marker data to demonstrate a measure of an average genome-wide LD block sizes. A pairwise LD values (*r* ^2^) are plotted against a genetic distance. Inner fitted trend line is a nonlinear logarithmic regression curve of *r* ^2^ on genetic distance. LD decay is considered below *r* ^2^ = 0.1 threshold and based on trend line it is around 38–40 cM in above plot. A pairwise LD between unlinked marker loci is assigned to 100 cM distance point. Note: this is for demonstration purposes only and does not have any real impact or correspond to any genomic fragment of an organism.

To estimate the size of these LD blocks, the *r* ^2^ values (alternatively, *D*′ can also be used) usually plotted against the genetic (cM) or weighted (bp) distance referred to as a “LD decay plot” (Figure 3). One can estimate an average genome-wide decay of LD by plotting LD values obtained from a data set covering an entire genome (i.e., with more or less evenly spaced markers across all chromosomes in a genome) against distance. Alternatively, the extent of LD for particular region (gene or chromosome) can be estimated from an LD decay plot generated using dataset obtained from a region of interest. When such a LD decay plot generated, usual practice is to look for distance point where LD value (*r* ^2^) decreases below 0.1 or half strength of *D*′ (*D*′ = 0.5) based on curve of nonlinear logarithmic trend line (see, e.g., [33, 43, 44]). This gives the rough estimates of the extent of LD for association study, but for more accurate estimates, highly significant threshold LD values (*r* ^2^ ≥ 0.2) are also used as a cutoff point. The decrease of the LD within the genetic distance indicates that the portion of LD is conserved with linkage and proportional to recombination [22, 25].

### 4.3. Factors affecting LD and association mapping

There are many genetic and demographic factors that play a role in the shaping of the haplotypic LD blocks in a genome [9, 22, 23, 25, 26]. Although mutation and recombination are one of the strong impact factors influencing LD [24], generally, factors affecting LD can be grouped into two categories: (1) factors that increasing LD, and (2) factors that decreasing LD. The increase of LD is observed with new mutation, mating system (self-pollination), genetic isolation, population structure, relatedness (kinship), small founder population size or genetic drift, admixture, selection (natural, artificial, and balancing), epistasis, and genomic rearrangements [25, 26]. The decrease of LD is observed with high recombination and mutation rate, recurrent mutations, outcrossing, and gene conversions [25, 26].

LD conserved with linkage is very useful for association mapping. However, more often there is a significant LD between pairs of loci located far from each other or even in different chromosomes that might cause spurious correlations in association mapping. These long stretched LD or LD between unlinked loci indicate the existence of other LD generating factors than linkage itself in a genome [9, 22, 23]. One of those factors is selection that generate LD between unlinked loci through “a hitchhiking” effect (high-frequency sweeping and fixation of alleles flanking a favored variant) [45], and epistatic selection or the so-called coadapted genes [46] that is the result of coselection of loci during breeding for multiple traits [26], common in traditional crop breeding programs worldwide.

The population structure (existences of distinctly clustered subdivisions in a population) and population admixture are the main factors to create such an LD between unlinked loci. This primarily happens due to the occurrence of distinct allele frequencies with different ancestry in an admixed or structured population. Theoretically, relatedness generates LD between linked loci, yet it might also generate LD between unlinked loci pairs when predominant parents exist in germplasm groups. There is evidence that relatedness caused LD between linked and unlinked loci in an equal proportion in maize germplasm [22]. The high ratio value of linked to unlinked loci in LD is good indicative to draw conclusion about the role of LD generating factor(s) such as selection or population stratification (cryptic relatedness) [9, 22, 23]. The other factors such as genetic drift or bottlenecks might have also generated LD in a genome [22–24], which is evidenced by nonuniform distribution of LD in chromosomes [24].

Knowing these factors that are increasing or decreasing LD in a genome, obvious question one might ask is whether increased or decreased level of LD is favored in association mapping? Very extensive level of LD (means LD persists within a long distance), theoretically, reduces a number of markers needed for association mapping, but makes resolution lower (coarse mapping). In contrast, less extensive level of LD (means that LD quickly decays within a short distance) requires many markers to tag a gene of interest, but in high resolution (fine mapping). Hence, choosing a population with low or high level of LD depends on the objective of association mapping study. Furthermore, increased LD level due to LD between unlinked loci is not salutary in association mapping since it tends to cause spurious marker-trait associations. LD generated by selection, population structure, relatedness, and genetic drift might be theoretically useful for association mapping in specific situations and population groups that reduces number of markers needed for association mapping [9, 22], but requires serious attention to control factors affecting LD (e.g., population structure and relatedness) to perform unbiased population-based association mapping in plants [41, 47] (see next sections).

There are other factors affecting LD referred to as a whole “ascertainment bias” that are associated with an assayed sample and data characteristics. Some of these factors leading to inaccurate estimate of LD were well reviewed by Gupta et al. [25]. One of such factors largely affecting the LD and leading inaccurate estimates is the presence of minor alleles (also referred as to rare alleles that are present in only 5 to 10% individuals of the sample) in a dataset. Minor alleles are problematic in LD quantification as they largely inflate LD values (in particular the *D*′ and *p*-values) [43, 48–50]. The *r* ^2^ is also very sensitive and has a large variance with rare alleles [43, 51]. Hence in the quantification of LD and association mapping, markers with minor allele frequency of 5–10% (varied from study to study) are (1) removed before analysis (see, e.g., [17, 18, 43, 44, 51]), (2) pooled into common allelic class (see, e.g.,[44, 46]), and (3) replaced with missing values (see, e.g., [52, 53]).

### 4.4. Estimation of LD using dominant versus codominant markers

The quantification methodology of LD, perfectly suitable for biallelic codominant type of markers (majorly, single nucleotide polymorphisms (SNPs) and now largely extended to multiallelic simple sequence repeats-SSRs), has been well developed and used in human, animal, and plant populations (for reviews see [25, 27–30]). LD quantification using dominant markers (such as random amplified polymorphic DNAs-RAPD, and amplified fragment length polymorphisms-AFLPs) is poorly explored and usually subject to wrong perception and interpretation. However, many underrepresented plant species, like forest trees, or other crops with limited genomic information largely rely on dominant type of markers such as RAPDs and AFLPs [54]. Furthermore, even with codominant, and multiallelic SSR markers, there is a great challenge with assigning correct allelic relationships (identity by decent) of multiple band amplicons when diverse, reticulated, and polyploid germplasm resources, lacking historical pedigree information, are genotyped. Misassignment of allelic relationships of loci is the concern in association analysis [55]. To avoid such a challenging cases, (1) one might select only single band SSR loci and code a dataset as a codominant marker type, yet such a single band SSRs are usually not many in polyploid crop genomes and yield also multiple bands when very diverse germplasm resources are genotyped; (2) alternatively, multiple-band SSRs with unknown allelic relationship may be scored as a dominant marker taking each band as an independent marker locus (uniquely) with a clear size band separation (see, e.g., [52, 56]).

Could a dominant marker data be used for LD quantification? There are some reports where LD level of natural forest tree populations has been measured using dominant markers (AFLPs) and commonly used statistical approach (see, e.g., [57]). There are also a number of reports where dominantly coded (present versus absent) marker data of RAPD, RFLP, AFLP, “candidate gene” (CAPs), and SSRs were successfully used in genome-wide LD analyses and LD-based association mapping in plants (see, e.g., [17–19, 56, 58, 59, 60]), demonstrating the feasibility of dominantly coded molecular data in revealing of haplotypic associations. Although a dominant type of coding has limited statistical power compared to codominant markers in population-based analyses because of missing heterozygote information, previous studies suggested that it can be successfully applied to the clustering of individuals and grouping of populations using a Bayesian approach when a large number of loci are genotyped [61, 62]. Dominant-type markers can be a useful tool to estimate the kinship coefficients between individuals [63].

Recently, Li et al. [54] investigated the use of dominant markers in estimation of LD in diploid species and developed appropriate EM algorithm. Based on their conclusion from the comparative data simulation of dominant versus codominant markers, the dominant-type markers could effectively be used in LD analysis with preferentially large number of marker loci and population sample sizes of ≥200 for high heterozygous (proportion of alternative alleles (present versus absent) in a data set, i.e., 0.5 versus 0.5) marker data or with even larger sample size ≥400 for low-heterozygous (i.e., 0.9 versus 0.1) dominant markers. It is also recommended that a mixture of codominant and dominant markers should be used to better characterization of a genetic structure of a population [54].

### 4.5. LD quantification in plants

LD quantification and LD-based association mapping have been a research objective in plants beginning with the model organism as Arabidopsis, and now extended to crops as maize, barley, durum wheat, spring wheat, rice, sorghum, sugarcane, sugar beet, soybean, and grape, as well as in forest tree species, and forage grasses.

Nordborg et al. [20] sequenced 0.5–1 kb long 13 fragments from a 250 kb region surrounding the flowering time *FRI* gene in a 20 global sample of *A. thaliana*, highly selfing model plant species. They determined that LD decays within a 1 cM distance or 250 kb. Later, investigation of the same authors [21] with markers surrounding the disease resistance locus *RPM1* in a globally-derived set of 96 Arabidopsis accessions revealed that a genome-wide LD extended up to 50–250 kb. LD blocks extended up to 50–100 cM in local Michigan Arabidopsis populations. These long-stretched LDs in local Arabidopsis population were explained as a genetic bottleneck or founder effect through introduction *A. thaliana* into North America in recent past (200 years ago). In contrast, in other study that targeted the region surrounding another disease resistance gene *rps5*, Tian et al. [64] reported much smaller LD block size, extended up to only 10 kb. Likewise, LD quickly decays within 10–50 kb distance around the *CLAVATA* 2 region of Arabidopsis [65]. Recently, Ehrenreich et al. [66] reported the LD decay within ~10 kb in extensive sequence analysis of 600-bp fragments of the regions *MORE AXILLARY GROWTH 2* (*MAX2*) and *MORE AXILLARY GROWTH 3* (*MAX3*) in a panel of 96 accessions from a restricted geographic range in Central Europe. In their genome-wide survey of 1347 fragments of 600-bp lengths, Plagnol et al. [67] reported that LD completely disappears after ~100 kb, which is comparable to that observed in human.

In maize (*Zea mays* L.), a highly outcrossing crop species, very rapid genome-wide LD decay was determined. Tenaillon et al. [68] first reported the extent of LD for maize, genotyping of 21 loci of chromosome 1 over the 25 individuals of the exotic landrace and United States maize germplasm. An average LD decay was determined to occur within 400 bp with *r* ^2^ = 0.2 and extended up to 1000 bp (~1 kb) in a group of US inbred lines. Later, Remington et al. [43] also reported a very rapid decline of LD in their survey of 6 genes (1.2–10 kb long) in 102 inbred lines, including tropical and semitropical lines with a wide genetic diversity. For these surveyed genes, LD declined generally within 200–2000 bp with *r* ^2^ = 0.1 except *sugary1* (*su1*) loci, where LD remained significant (*r* ^2^ = 0.3 − 0.4) for 10 kb distance. This was explained by strong selective episodes in *su1* gene. In the same study, Remington et al. [43] found higher level of LD with 47 SSR markers compared to those obtained from SNP data. This result was explained by different mutation rate of these two marker systems that tends to capture different historic information.

Long stretches of LD for maize also were reported. Thornsberry et al. [69] measured LD in and around the *Dwarf8* locus. They found “localized LD” (i.e., restricted to particular regions, meaning that high LD stretches interspersed with regions of low LD) extended up to 3 kb. Jung et al. [70] reported the extent of LD within 500 kb in surveying *adh1* locus. Stich et al. [22] examined the genetic diversity and LD in a cross section of 147 European and United States elite inbred material with 100 SSRs. They reported an average significant (*P* < .5) LD block size of 26 cM for flint group, or 41 cM for dent group with nonuniform distribution of LD among 10 chromosomes. They showed a very long stretched LD blocks up to 105 cM in chromosome 2 and up to 103 cM in chromosome 7 in flint and dent groups, respectively. Obtaining of different result from earlier studies [43] was explained due to using (1) much higher marker density, and (2) both related and unrelated inbred lines. In another study, the same authors [9] examined 72 European elite inbred lines with 452 AFLP and 93 SSR markers and reported much shorter average LD block sizes for AFLP (4 cM), but extensive LD for SSR (30-31 cM) in both flint and dent germplasm groups. This suggested a potential for exploiting both markers in association mapping, but with the favor of SSRs over AFLPs because of power of detecting LD. Recently, Andersen et al. [71] reported that LD is persisted over entire 3.5 kb *phenylalanine ammonia lyase* (*PAL*) gene with the *r* ^2^ > 0.2 in a survey of 32 European maize inbred lines.

In the selfing tetraploid wheat (*Triticum durum* Desf.), Maccaferri et al. [50] quantified LD in a 134 durum wheat accessions that extended up to 10 and 20 cM with *D*′ = 0.67 and 0.43, respectively. In hexaploid wheat (*Triticum aestivum* L), almost completely self-pollinating species, strong LD was determined to occur on average within <1 and ~5 cM for region on chromosome 2D and centromeric region 5A that was surveyed with 36 SSR markers in a 95 cultivars of winter wheat [52]. Recently, Chao et al. [72] investigated the genome-wide LD among 43 US wheat elite cultivars and breeding lines representing seven US wheat market classes using 242 SSRs distributed throughout the wheat genome. For this germplasm collection, a genome-wide LD estimates were generally less than 1 cM for the genetically linked loci pairs. Most of the LD regions observed were between loci less than 10 cM apart, suggesting LD is likely to vary widely among wheat populations [72]. Tommasini et al. [56] reported that LD on chromosome 3B extended up to 0.5 cM in 44 varieties or 30 cM in 240 RIL populations of winter wheat, surveyed with 91 SSR and STS markers. This suggested usefulness of cultivar germplasm over biparental mapping population in association mapping.

In rice (*Oryza staiva* L), a selfing species, Garris et al. [73] examined the LD surrounding disease resistance locus *Xa5* using 21 SSRs in a survey of 114 rice accessions. They determined the strong LD within 100 kb with *r* ^2^ = 0.1. Agrama and Eizenga [74] investigated LD patterns in a worldwide collection of *Oryza staiva*, and its wild relatives using 176 SSR markers. Although it was not specifically indicated, LD decay plot suggests a long range LD declining ~50 cM with *D*′ = 0.5 in the “International” and “US” rice collections. Interestingly, LD persisted over an average of 225 cM distance with significant *D*′ > 0.5 in a wild accessions. In contrast, many other studies reported a less extent of LD in wild and landrace (broad-based) germplasm and high extent of LD in cultivar (narrow-based) germplasm resources in plants [9, 43]. There is evidence that the LD is remarkably different in other rice species. Rakshit et al. [75] reported that LD in *O*. *rufipogon* decays within 5 kb, while it declines at 50 kb in *O. sativa* ssp. *indica* accessions. Mather et al. [76] observed that the extent of LD is greatest in temperate *japonica* (>500 kb), followed by tropical *japonica* (~150 kb) and *indica* (~75 kb) that was revealed by using unlinked SNPs. LD extends over a shorter distance in *O. rufipogon* (≪40 kb) than in any of the *O. sativa* groups assayed in their study [76].

LD also has been extensively quantified another highly self-pollinated crop, barley (*Hordeum vulgare* L), where the extent of LD varied from 10 cM to 50 cM range depending on assayed set of a germplasm [17, 77]. Caldwell et al. [51] measured LD in four genes surrounding hardness locus (*Ha*) in three different gene pools and reported a long stretched LD extended up to at least 212 kb in inbred barley and 98 kb in landrace barley germplasm. In contrast to these long range LDs observed in barley germplasm, Morrell et al. [78] reported a rapid decay of LD detected within 300 bp in their study of 18 nuclear genes (average length of 1 361.1 bp) in 25 diverse wild barley accessions. In that, LD completely disappeared within a 1200 bp distance. This demonstrates another example of variability of LD quantification across germplasm resources, breeding material, and regions tested.

Furthermore, genome-wide LD has been quantified for many other plant species that extended up to 10 cM in sugar cane (*Saccharum*) [10], 10–50 cM in soybean (*Glycine max*) [79, 80], 3 cM in sugar beet (*Beta vulgaris* L) [81], 50 cM in sorghum (*Sorghum bicolor*) [44], 5–10 cM in grape (*Vitis vinifera* L) [53], 16–34 kb in poplar (*Populus trichocarpa*) [82], <500 bp in European aspen (*Poplus termula*) [83], 2000 bp in loblolly pine (*Pinus taeda*) [84], 1000 bp in Douglas-fir (*Pseudostuga menziensii*) [85], 100–200 bp in Norway Spruce (*Picea abies*) [86], 200-1, 200 bp in silage maize (*Zea mays L*) [87, 88], and 500–2000 bp in ryegrass (*Lolium perenne*) [89–91]. Also, LD quantification for other important crops, perhaps, is in progress. In this context, recently, we have quantified LD level for improved varieties and landrace stock germplasm of cotton (*Gossypium hirsutum* L) [92]. Survey of 200 microsatellite markers in 335 *G. hirsutum* variety germplasm demonstrated that a genome-wide averages of LD extended up to genetic distance of 25 cM with *r* ^2^ > 0.1. Likewise, our another companion study using 95 core set microsatellite markers in a total of 286 “exotic” *G. hirsutum* revealed that a genome-wide averages of LD decays within the genetic distance at <10 cM in the landrace stocks germplasm and >30 cM in photoperiodic variety germplasm, providing evidence of the potential for association mapping of agronomically important traits in cotton (Abdurakhmonov et al. unpublished, submitted elsewhere for publication).

### 4.6. Implications for association mapping gained from LD quantification studies in plants

Important information and implication for association mapping gained from above studies are that: (1) LD more quickly declines in outcrossing plant species than highly self-pollinating plants, enabling high resolution mapping of a trait of interest in outbreeder plant germplasm. At the same time, LD rapidly declines in crop variety groups (even in selfing species) compared to populations derived from biparental crosses, which provides an advantage of discovery more polymorphisms in the variety germplasms than biparental populations of self-pollinated crops [56]; (2) the extent of LD varies across the genomic regions, among population samples and between species with the examples of “localized LD”; (3) LD measures differ per marker systems used as a reflection of capturing of different historic information in a genome due to different mutation rate (e.g., SNP versus SSR or AFLP versus SSR); (4) an estimate of genome-wide averages for the extent of LD in plant germplasm may not adequately reflect LD patterns of specific regions or specific population groups. Each of these specific regions or population groups should additionally be explored for the extent of LD in order to conduct successful association mapping of variants within regions or populations of interest; (5) LD blocks in narrow-based germplasm groups are longer than broad-based germplasm groups in plants [9, 43]. This suggests an opportunity perform coarse mapping with less number of markers in narrow-based plant germplasm and then fine mapping in broad-based plant germplasm, assuming that genetic causations is sufficiently similar across germplasm groups [12]. This also suggest an opportunity develop a set of mapping populations with the required amount of LD and diversity for high-resolution mapping through directed crossing between selected broad- and narrow-based germplasm groups [86]; and (6) confounding population characteristics and biological behavior have serious impact on pattern and structure of LD in plant germplasm resources that need to be taken into consideration in conducting unbiased association mapping.

## 5. ASSOCIATION STUDIES IN PLANTS

### 5.1. The methodology overview

There are many types of different methodologies that have been developed and initially are widely used for association mapping studies in human (comprehensively reviewed by Schulze and McMahon [93]), yet perfectly applicable without change or case-to-case modifications for wide range of organisms, including plants. Lately, some considerably successful achievements have been made to develop powerful, more precise, and unbiased population-based association-mapping methodology for plants. Here, we provide a brief overview for a basic concept and ideology of widely used pioneer methodologies for association mapping, and then highlight the latest developments in the methodology and experimental design of association mapping in plant population with the examples of association mapping of useful traits in crop species.

The classical methodology and design of association mapping is “case and control” (also referred to as “case-control”) approach that identifies the causative gene tags in the comparison of allele frequencies in a sample of unrelated affected (referred to as “cases”) individuals and a sample of uninfected or healthy individuals (referred to as “controls”) [93, 94]. This design requires an equal numbers of unrelated and unstructured “case-control” samples for accurate mapping. The Pearson chi-square test, Fisher's exact test, or Yates continuity correction can be used for a comparison of allele frequencies and detection of an association between a disease phenotype and marker. Although favored, the random sampling individuals from a population do not provide the equal representation of case and controls in the mapping population since cases in the population are usually low, thus requires special efforts to collect the cases. Case and control approach is seriously affected by the existence of population structure and stratification that caught the attention of scientist [93]. Falk and Rubinstein [95] developed a haplotype relative risk (HRR) approach that minimizes, but not eliminates population stratification issues in association mapping [96]. In that, first, a “pseudocontrol” group (containing combination of two alleles that are not transmitted to affected offspring) is created; then, the marker allele frequencies in case and “pseudocontrol” groups are correlated [93].

To efficiently eliminate the confounding effects coming from population structure and stratification, Spielman et al. [97] developed transmission disequilibrium test (TDT) method that compares transmission versus nontransmission of marker alleles to affected offspring by using chi-square test [93], assuming a linkage between marker and trait. The TDT design requires genotyping of markers from three individuals: one heterozygous parent, one homozygous parent, and one affected offspring. Although HRR performs better with unstructured sample than TDT because of its power to completely eliminate spurious association with good experimental design, later is widely used as a tool for unbiased fine mapping of traits in the presence of linkage with a biallelic, one marker model that can accommodate pedigree structure [30, 93].

Nonetheless, initial TDT approach had issues with the use of multiallelic markers, multiple markers, missing parental information, extended (larger) pedigrees, and complex quantitative traits [93]. To address these issues, a variety of extensions of TDT approach were developed and applied for multiallelic markers (i.e., GTDT, ETDT, MC-Tm) [98–102], multiple markers [103–105], missing parental information (Curtis-test, S-TDT, SDT, 1-TDT, C-TDT or RC-TDT) [96, 106–110], which were reviewed by Schulze and McMahon [93] in detail. Shortly after publication of various extensions of TDT to multiallelic and multiple markers, the extensions for X-linked genes, such as XS-TDT or XRC-TDT were developed and applied [111]. TDT approach was also extended to pedigrees of any size as a PDT approach [112, 113] that was demonstrated more powerful than TDT, and S-TDT or SDT under the assumption of high disease prevalence [93, 114].

Further, there were many studies to extend the TDT approaches to QTL and covariates [93]. One of the comprehensive approaches, QTDT was developed with its three different extensions for quantitative traits for any pedigree structure [115, 116]. These family-based association-mapping approaches have their other improvements using more powerful statistical and robust algorithmic procedures, such as likelihood-base statistics and EM algorithm (TDTLIKE, LRT, EM-LRT) [117–119]. The unified family-based association test package (FBAT) incorporating some of TDT is also developed [120–122] to deal with wide types of experimental designs. The next generation of association mapping approaches in both “case and control” and family-based designs, referred to as identity by decent (IBD) mapping [123], haplotypes-sharing analysis (HSA) [124], and decay of haplotypes sharing (DHS) [125], involves the analysis of haplotypes by testing the length of haplotypes in the data sample, assuming affected individuals will have a longer haplotypes than controls [93].

Although family-based association mapping methodology is effective to control confounding effects of a sample and remove spurious associations, it is less powerful design [126] and have its disadvantageous sides compared to case-control [93] that led to develop the methodologies with better controlling of population structure and stratification. Such an improved methodology for a case and control design or random samples from a population involves the use of additional markers that have neutral effect (null loci) to the trait of interest in the analysis. This approach is referred to as the genomic control (GC) that finds confounding effects of a population and corrects it, thus enabling to remove spurious associations [127, 128]. Although GC is powerful then TDT [128], it will not remove spurious associations in highly structured populations. Zhao et al. [129] put it as 

“*Methods like* “*genomic control*,” *which simply rescale p-values without changing the ranking of loci are not likely to be useful in genome-wide scans where the existence of true positives is not in doubt*.”

To better deal with highly structured populations, Pritchard et al. [47, 62] developed approach of structured association (SA). SA first searches a population for closely related clusters/subdivisions using Bayesian approach, and then uses the clustering matrices (*Q*) in association mapping (by a logistic regression) to correct the false associations. Population structure and shared coancestry coefficients between individuals of subdivisions of a population can be effectively estimated with *STRUCTURE* program using several models for linked and unlinked markers [130]. Similar type of methodology measuring and using the population subdivisions (*K*) in association mapping referred to as “mixture model” was proposed by several other studies [131, 132]. However, SA incorporating only population structure information in the analysis is not good enough itself when highly structured population with some degree of related individuals used in the association mapping.

Hence, recently, Yu et al. [133] developed new methodology, a mixed linear model (MLM) that combines both population structure information (*Q*-matrix) and level of pairwise relatedness coefficients—“kinship” (*K*-matrix) in the analysis. To perform MLM: (1) *Q*-matrix is generated using *STRUCTURE*, (2) the pairwise relatedness coefficients between individuals of a mapping population (*K*-matrix) [134] measured using *SpaGedi* software [135], and (3) then both *Q*- and *K*-matrices are used in association mapping to control spurious associations. Although computationally intensive, MLM approach found to be effective in removing the confounding effects of the population in association mapping [133].

Later Zhao et al. [129] extensively tested the MLM approach of Yu et al. [133] in their global set of 95 highly structured Arabidopsis population and came to overall agreement with better performance of *Q* + *K* MLM model than any of the other tests that used *K*- or *Q*-matrix alone. However, they also noted that (1) *K* matrix would alone be good enough if a kinship estimated as a proportion of shared haplotypes for each pair of individuals (as denoted K*); (2) the replacement of *Q*-matrix (from the computational intensive *structure* analysis) [130] with *P*-matrix (from more robust principal component analysis) [136] performed similarly to MLM of Yu et al. [133], thus suggesting a potential for future replacements; (3) removing of the confounding effects will also subject to remove true associations with biological effect, which is strongly correlated with population structure that requires a caution; and (4) in a small and highly structured population, the causations with major effect should be expected to be found and, perhaps, larger samples and adequate marker densities are needed for genome-wide dissection of the most traits of interest segregating in an association mapping population [129].

There are other types of mixed models for association mapping that have its own advantages to control population confounding effects and tag a genetic causative of a trait of interest. One of such mixed models utilizes a sample with pedigree information to measure a pedigree-based relatedness and incorporates it directly in QTL-mapping and association mapping [59, 137, 138]. This type of mixed model for known pedigree population combines haplotype effects with pedigree-based structure of variance-covariance relatedness matrix and random polygenic effect that control the population structure [59, 139]. The efficiency of pedigree population for association mapping depends on the population size of pedigree founders (i.e., pedigree population obtained from just two parents will not provide significant level of LD) and the level of relatedness of the founders. Latter is very important and may still lead to spurious association due to initial population structure (mostly unknown) coming from founders that needs to be analyzed also by using *STRUCTURE* [140].

However, as stated by Malosetti et al. [59] and others [140] the pedigree-based mixed model is highly appropriate in association mapping in crops due to (1) plant breeding programs have already generated many useful pedigree populations that contain LD useful for association studies but cannot be used as an independent LD-mapping population, and (2) many historical trait data sets in plant breeding are unbalanced that have been collected over multiple-years, and multienvironmental trials. At the same time, issues with obtaining the fine-grained pedigree information and difficulty of finding population structure of narrow-based elite cultivars are the concern in pedigree-based mixed model. There is another mixed model that combines the Bayesian variable selection for mapping multiple QTLs and LD mapping method, incorporating estimates of population structure, but not relatedness. This approach was used for association mapping in highly selfing rice germplasm [58]. Authors stated that incorporation of multiple QTL effects and population structure efficiently reduces spurious association and useful for future whole genome associations, with the development of more complex models dealing with differences of LD and effect of QTL alleles between populations.

The other mixed model approach combines QTL and LD analyses of distinct studies. In that, QTLs or candidate genes with already annotated biological function(s) are used as a priori information in association mapping [140, 141]. This is one of the effective alternative strategies in association mapping that allow reducing the total amount of marker genotyping (because of preselecting of markers restricted to QTL region) in less number of individuals. This increases the power and precision of the trait-marker correlations [142].

### 5.2. Power of association mapping

The power of association mapping is the probability of detecting the true associations within the mapping population size that really depends on (1) the extent and evolution of the LD in a population, (2) the complexity and mode of gene action of the trait of interest, (3) sample size and experimental design. The power can be increased utilizing the better data (knowledgeable experimental design and accurate measurements) and increasing the sample size. In QTL mapping studies, there are specific statistical approaches to estimate the false-positive level of the obtained strong (*p*-value) associations (control for Type I error) such as a permutation test [143] or false discovery rate (FDR) [144].

A statistical approach within the Bayesian framework is used test the reliability of obtained significance (*p*-values) in association mapping because of possibility of getting unreliable values due to (1) overestimation of effects (selection bias), (2) association coming from neglecting confounding effects of a sample, (3) poor experimental design, and (4) instability of genetic effects across different environments [142]. Ball [142] developed a methodology, combining the Bayesian and non-Bayesian approaches, that determines the *Bayes* factors guiding to properly design the experiments with given power to detect reliable effects. To detect the reliable effects in association mapping, experiments should be designed at least with the *Bayes* factor of 20 that may require much larger sample sizes. *Bayes* factor provides stronger evidence than conventional *p*-values [142]. If given *Bayes* factor value (say B = 20) reached with larger sample than the original experimental design, then, the original results indicate a very weak evidence to provide the real effects [142]. At this point, requirement for larger sample size might make association mapping disadvantageous over a traditional QTL-mapping. However, the sample size for association mapping can be decreased keeping the high power with (1) preselecting a priori known QTL regions or candidate genes (from QTL-mapping and expression analyses), (2) using the large populations with samples longer LD block that require a less number of markers to find useful associations, (3) an alternative experimental design (i.e., TDT), and (4) choosing the single marker from the haplotypes of interest that would cut also marker number and so genotyping cost [142]. *Bayes* factor can be calculated using *R* function of *ld.design* from *ldDesign* package [140]

### 5.3. Examples from reports

The pioneer association studies in plants were performed by Beer et al. [166] in oat, and by Virk et al. [167] in rice. Beer et al. [166] associated 13 QTL with RFLP loci using 64 oat varieties and landraces, yet without considering the population structure that resulted in more increased associations than what were obtained in separate analysis of subpopulations [11]. Virk et al. [167] predicted 6 trait values using RAPD markers in rice germplasm. Later, association mapping was extended to sea beet, barley, maize, wheat, potato, more examples in rice, and Arabidopsis that have utilized population level of LD considering a population structure. Hansen et al. [19] reported association of ALFP markers with bolting gene in sea beat. In barley, various traits such as yield, yield stability, heading date, flowering time, plant height, rachilla length, resistance to mildew and leaf rust were associated with many different types of molecular markers [17, 18, 157, 158]. In maize, flowering time and plant height [43, 69] were associated using SNP and SSRs. Following these pioneer studies of association mapping in maize, several other traits such as phenotypic variation in flowering time, endosperm color, starch production, maysin and chlorogenic acid accumulation, cell wall digestibility, and forage quality were associated using SNP markers of candidate genes [71, 87, 88, 149–153].

In wheat, Breseghello and Sorrells [52] reported first association mapping of kernel size and milling quality in a collection of USA winter wheat using SSRs. Following this work, association mapping of a high molecular-weight glutenin [159] and blotch resistance [56] were reported that utilized SNPs, SSRs, and STS markers. In rice, association mapping has not widely been applied yet due to highly structured population of rice (due to high selfing) [58, 133]. However, Zhang et al. [156] successfully used association mapping for multiple agronomic traits using discriminant analysis (DA) with SSR and AFLP markers. Recently, Iwata et al. [58] associated RFLP markers with width and length of milled rice grains in a set of 332 rice germplasm using their multiple QTL model considering the population structure. Association mapping approach was also successfully applied in tetraploid potato where resistance to wilt disease [138], bacterial blight [60], *Phytophtora* [59] that utilized a pedigree-based mixed model.

To date association mapping has also been extended to long lifespan plant species, forest tree populations [163], where associations of polymorphisms in *cinnamoil CoA reductase* (*CCR*) with earlywood microfibril angle trait [141], and polymorphisms a putative stress response gene with wood density and wood growth rate [163] were reported. There are also the examples of association mapping successes for cold tolerance, flowering time, water-soluble carbohydrate content, and forage quality in forges species that have recently been reviewed by Dobrowolski and Forster (Table 1) [87, 88, 161].

Association mapping of traits in Arabidopsis also has been reported and overall suitability of the approach well documented. Associations of *CRY2* with flowering time were reported [145, 146]. Balasubramanian et al. [147] reported the association of *PHYC* with flowering and growth response in Arabidopsis. Later Zhao et al. [129] revisited to these association results with their mixed model approach and reproved some of previously reported associations (with *PHYC*), but challenged the power of these associations detected by using “standard linear methods without correcting population structure.” They put it as “*Clearly, none of these polymorphisms would have been picked up in a genome-wide scan*” while noting the use of different sample and trait measurements in the original studies. They also reported one of the significant flowering time associated polymorphisms in *CLF* gene in their genome-wide analysis using MLM [129]. Flowering time (in *FRI* gene) and pathogen resistance (in *Rpm1*, *Rps5*, and *Rps2* genes) associated polymorphisms were also reported [148]. Recently, Ehrenreich et al. [66] reported polymorphisms of candidate genes (*SPS1*, *MAX2*, and *MAX3*) associated with branching architecture in a survey of 36 genes involved in branch development that were genotyped in a panel of 96 Arabidopsis accessions from Central Europe.

### 5.4. Choice of the appropriate approach

Table 1 summarizes the LD and association mapping efforts in plants including some of very recent whole genome association mapping studies. As one can see, within the frame of above highlighted association studies in plants, various association mapping methodologies (Table 1), molecular markers (both dominant and co-dominant markers), and plant germplasm resources (including landrace stocks, elite germplasm, and experimental populations—e.g., RILs) have been utilized. Identifying of the most appropriate approach and marker systems, therefore, is challenging and might be irrelevant case-to-case basis.

Choosing the appropriate association mapping depends on (1) the extent and evolution of the linkage disequilibrium in a population, (2) the level of population structure and stratification, (3) availability of pedigree information, (4) complexity of the trait of interest under study, and (5) availability of the genomic information and resources. Based on reported studies, GC is favored approach when population structure is suspected, but failed to be detected [59]; however, MLM considering both relatedness and population structure [133] and pedigree-based mixed model [59] or multiple QTL model [58] performs well in most cases with highly structured and stratified population although one still might argue based on his own experience, knowledge, and type of gemplasm used. According to Stich et al. [23], SA and MLM models do not “explicitly correct” for LD caused by selection and genetic drift, the major factors causing LD in plant germplasm and breeding materials. Hence Stich et al. [23] suggested use of family based association approach [168] with breeding materials. However, again the choice of methodology greatly depends on the germplasm used for mapping. The germplasm materials used for association mapping were comprehensively discussed by Breseghello and Sorrells [169].

## 6. CONCLUSIONS

Thus the association mapping methodology, initially developed by the human geneticists, has found its successive application in plant germplasm resources, in particular after recent improvements in minimization of spurious associations. The examples of association mapping studies performed in various plant germplasm resources including model plant Arabidopsis and extended to various crop germplasm largely demonstrate the flourish of crop genomics era with the utilization of powerful LD-based association mapping tool. This is also a good indicative of the potential utilization of this technology with the other crops and plant species in the future. Currently, a number of such studies are, perhaps, in progress in many laboratories worldwide. The near-future completion of genome sequencing projects of crop species, powered with more cost-effective sequencing technologies, will certainly create a basis for application of whole genome-association studies [164], accounting for rare and common copy number variants (CNV) (for review see, e.g., [165]) and epigenomics details of the trait of interest in plants, which is widely being applied in human genetics with great success. This will provide with more powerful association mapping tool(s) for crop breeding and genomics programs in tagging true functional associations conditioning genetic diversities, and consequently, its effective utilization.

## Figures and Tables

**Table 1:** Linkage disequilibrium and association mapping studies in plants.

Species	Mating system	LD extent	Mapped traits	*Approach used
Arabidopsis	Selfing	10–250 kb and 50–100 cM [20, 21, 64, 66, 67]	Flowering time, growth response, pathogen resistance, and branching architecture [66, 129, 145–148]	One way ANOVA, simple regression, SA, MLM
Maize	Outcrossing	200–2000 bp [43, 68], 3–500 kb [43, 69–71], 4–41 cM [9, 22]	Plant height, flowering time, endosperm color, starch production, maysin and chlorogenic acid accumulation, cell wall digestibility, forage quality, and oleic acid level [43, 69, 71, 87, 88, 149–154]	GLM, SA, MLM, WGA
Rice (*indica, japonica* and *rufipogon*)	Selfing	5–500 kb [73, 75, 76] 50–225 cM [74], 20–30 cM [155]	Multiple agronomic traits such as plant height, heading date, flag leaf length and width, tiller number, steam diameter, panicle length, grain length and width, grain length/width ratio, grain thickness, 1000-grain weight, width and length of milled rice grains [58, 155, 156]	DA, MLM, mixed model with multiple QTL effect
Barley	Selfing	10–50 cM [16, 77], 98–500 kb [51], 300 bp [78]	Yield, yield stability, heading date, flowering time, plant height, rachilla length, resistance to mildew, and leaf rust were associated with many different types of molecular markers [17, 18, 157, 158]	Pearson correlation; regression, ANOVA
Tetraaploid wheat	Selfing	10 and 20 cM [50]	N/A	N/A
Hexaploid wheat	Selfing	<1–10 cM [52, 56, 72]	Kernel size and milling, a high molecular weight glutenin and blotch resistance [52, 56, 159]	GLM-*Q*, LMM
Potato	Selfing	0.3–1 cM [25, 60], 3 cM [160]	Resistance to wilt disease, bacterial blight, *Phytophtora*, and potato quality (tuber shape, flesh color, under water weight, maturity, and etc.)[59, 60, 138, 160]	Nonparametric Mann-Whitney U test, standard two sample *t*-test, GMM
Soybean	Selfing	10–50 cM [79, 80],	Seed protein content [80]	WGA
Sorghum	Outcrossing	50 cM [44]	N/A	N/A
Grape	Vegetative propagation	5–10 cM [53]	N/A	N/A
Sugarcane	Outcrossing/Vegetative propagation	10 cM [10]	N/A	N/A
Sugar beet	Outcrossing	3 cM [81]	N/A	N/A
Forage grasses (silage maize and ryegrass)	Outcrossing	200–2000 bp [87–91]	Cold tolerance, flowering time and forage quality, water-soluble carbohydrate content [87, 88, 161, 162]	Multiple linear regression; ANOVA
Forest trees (Norway spruce, Loblolly pine, poplar, European aspen, Douglas-fir)	Outcrossing	100–200 bp [86], ~500–2000 bp [83–85]	Early-wood microfibril angle trait, wood density and wood growth rate [141, 163]	ANOVA; combination of LD and QTL mapping

* MLM: mixed linear model [133]; GLM: general linear model without population structure [71]; GLM-*Q*: general linear model using population structure matrix (*Q*) or the least square solution to the fixed effects GLM [56]; DA: discriminant analysis [156]; SA-structured association [47]; LMM: linear mixed model [52]; WGA: whole genome association [154, 164, 165]; GMM: general mixed model [59]; ANOVA: analysis of variance test; N/A—not available (search of known major online library database as of December 2007).
